# Reductions in ventilator-associated events following implementation of a ventilator-associated pneumonia diagnostic stewardship intervention: A difference-in-difference study

**DOI:** 10.1017/ice.2025.10376

**Published:** 2026-03

**Authors:** Owen Albin, Zachary Garcia, Jonathan Troost, Andrew Weirauch, Krishna Rao, Kevin Thompson, Emily Stoneman, Keith Kaye

**Affiliations:** 1 Department of Internal Medicine, Division of Infectious Diseases, University of Michigan Medical School, Ann Arbor, MI, USA; 2 Michigan Institute for Clinical & Health Research, University of Michigan, Ann Arbor, MI, USA; 3 Department of Adult Respiratory Care, Michigan Medicine, Ann Arbor, MI, USA; 4 Department of Infection Prevention & Epidemiology, Michigan Medicine, Ann Arbor, MI, USA; 5 Department of Internal Medicine, Robert Wood Johnson Medical School, New Brunswick, NJ, USA

## Abstract

In this *post hoc* analysis of a quasi-experimental pilot/feasibility trial, a bundled diagnostic stewardship intervention safely reduced respiratory culturing rates without increasing ventilator-associated events (VAEs). Using difference-in-differences methodology, we observed a significant reduction in possible ventilator-associated pneumonia (PVAP) events, suggesting the intervention may reduce pneumonia overdiagnosis without compromising patient safety.

## Introduction

In the Diagnosis of Ventilator-Associated Pneumonia (DIVA) study (NCT05176353), a quasi-experimental pilot/feasibility trial, we evaluated the safety and effectiveness of a bundled diagnostic stewardship intervention (DSI) targeting the respiratory culturing pathway in mechanically ventilated patients (MVPs).^1^ The DSI consisted of three components: (1) clinical decision support to restrict unnecessary respiratory culture ordering, (2) guidance to preferentially use bronchoalveolar lavage (BAL) over endotracheal aspiration for specimen collection, and (3) modifications to laboratory reporting workflows that conditionally suppressed BAL culture result reporting for samples with ≤50% neutrophils in BAL fluid analysis. Implementation of the DSI was associated with significant reductions positive respiratory culture rates (79 vs 69 per 1,000 MVP-days, *p* = 0.02) and greater use of BAL specimens (14.5% vs 25.6%, *p* < 0.01), without adversely affecting patient safety.

During routine data monitoring, we observed an unexpected decline in ventilator-associated event (VAE) rates in study ICUs following DSI implementation (VAEs per 1,000 MVP-days: pre-intervention 15 [95% CI: 13–17] vs. post-intervention 10 [95% CI: 7–14], *p* = 0.05). VAEs, defined by the CDC’s National Healthcare Safety Network, are a tiered set of surveillance events based on objective criteria designed to identify potentially preventable complications of mechanical ventilation (Supplemental Materials).^2^ This result was hypothesis-generating but was limited by the single-arm study design and the absence of advanced methods (eg, models that accommodate clustering and zero-inflated count data) better suited for robust inference across ICUs.

To more rigorously assess the impact of the DSI on VAE incidence and subtype distribution, we conducted a post hoc difference-in-differences (DiD) analysis using robust methods for clustered and zero-inflated data.

## Methods

### Study setting and data collection

The DIVA study was performed at the University of Michigan Health System, a tertiary academic medical center with seven ICUs totaling >100 beds. We used Infection Prevention surveillance data to capture monthly VAE counts and ventilator-days per ICU over an 8-year period (2017–2025), including a 1-year DSI implementation phase (February 2022–February 2023). Post-intervention data (March 2023–2025) reflect usual practice after DSI withdrawal. Study ICUs consisted of two medical ICUs that participated in the original pilot/feasibility study and received the DSI, while the remaining five ICUs—comprising surgical, trauma/burn, and neurosurgical units—did not implement the intervention and served as non-study comparator units. All ICUs employed identical VAE prevention bundles and demonstrated comparable compliance with bundle components during the study period. The study was approved by the University of Michigan Institutional Review Board using a waiver of informed consent.

### Difference-in-difference methodology

To estimate the intervention’s effect on VAE incidence rates, we used a DiD approach, a quasi-experimental design that estimates the change in outcomes in an intervention group relative to a comparator group over time, adjusting for time-invariant confounding as well as shared secular time trends.^3^ In this context, the DiD estimator quantifies the extent to which VAE incidence rates changed in study ICUs relative to non-study ICUs during DSI implementation. We implemented this using the following model:

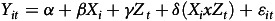




where X_i_ is an indicator for study ICU status, Z_t_ is an indicator for the intervention period, and δ is an interaction term (the DiD estimator) representing the estimated effect of the intervention on the outcome Y_it_. Prior to conducting analyses, we confirmed that key causal assumptions for the DiD framework—consistency, positivity, no interference, and parallel trends—were reasonably satisfied (see Supplemental Materials).

We calculated monthly VAE incidence rates as events per 1,000 MVP-days and visualized unadjusted trends using smoothed time series plots stratified by study ICU status. We initially considered mixed-effects and generalized estimating equation models; however, exploratory analyses demonstrated low within-cluster correlation and substantial zero inflation in event counts (see Supplementary Appendix). We therefore employed hurdle models for primary analyses.^4^ Hurdle models separately estimate the probability of any events and the count of events among non-zero observations. For possible ventilator-associated pneumonia (PVAP) events, where no events occurred in study ICUs during the intervention period, we used Firth’s penalized logistic regression to address separation and obtain a bias-reduced DiD estimate.^5^ All statistical analyses were performed in R version 4.4.2.^6^

## Results

**Figure 1.**
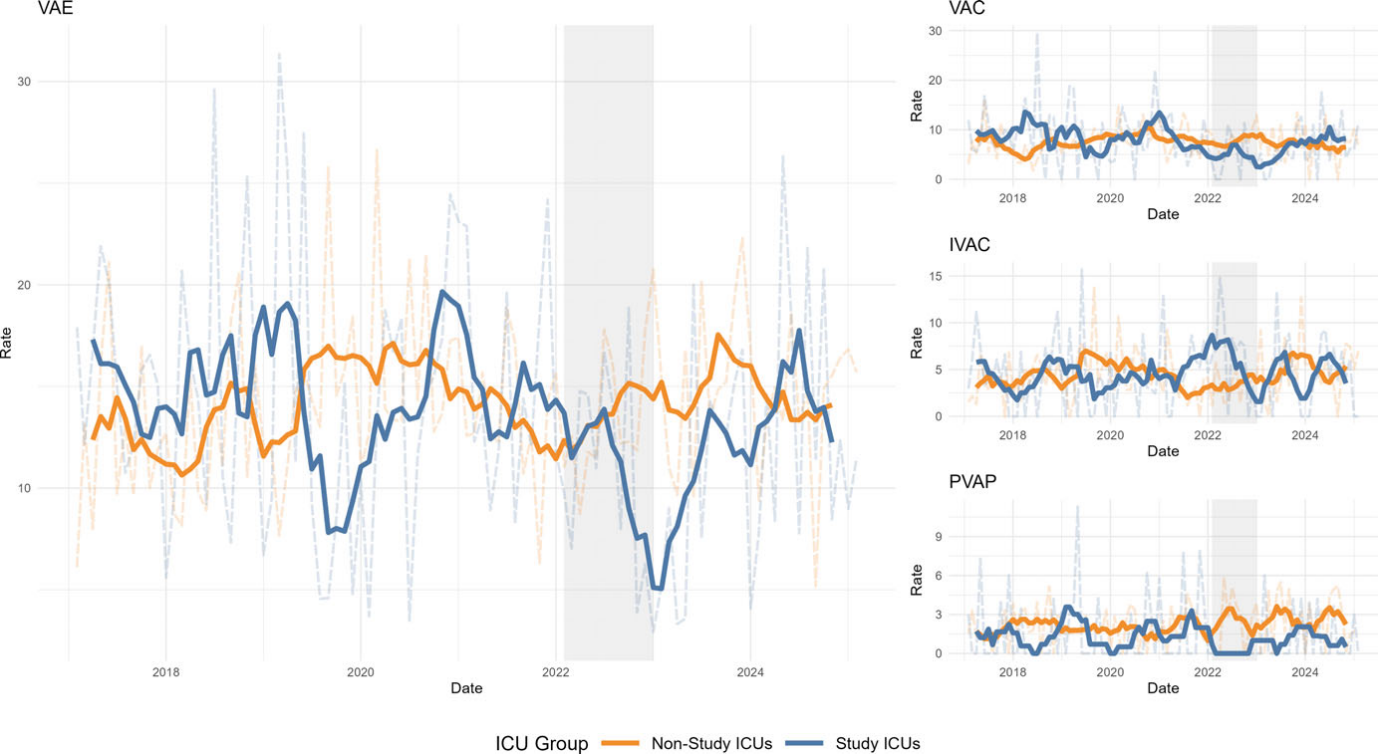
Time series of VAE incidence rates. Incidence rates expressed as number of monthly events per 1,000 mechanically ventilated patient days. Transparent dashed background lines represent monthly counts; solid lines represent a 6-month moving average. VAE, ventilator-associated event, VAC, ventilator-associated complication; IVAC, infection-related VAC; PVAP, possible VAP; Gray shaded area, study intervention period.

Trends in total VAE and VAE subtype incidence rates over time as a function of study ICU status are shown in Figure 1. Table 1 shows monthly VAE incidence rates and DiD estimates. During the intervention period, total VAE rates declined in study ICUs (from 14.3 to 10.1 events per 1,000 ventilator-days), while remaining stable in non-study ICUs. However, this difference did not reach statistical significance in either the zero model (OR = 0.60, 95% CI: 0.18–2.05; *p* = 0.42) or the count model (incidence rate ratio (IRR) = 0.68, 95% CI: 0.40–1.15; *p* = 0.15).

**Table 1.** VAE incidence rates and difference-in-difference estimates

ICU	Mean rate (non-intervention time period)	Mean rate (intervention time period)		DiD estimator(s)	
			Absolute change in mean rate	Zero-model OR (95% CI), *p*-value	Count model IRR (95% CI), *p*-value
**Total VAE**					
Study ICUs	14.3	10.1	**↓**4.2	0.60 (0.18–2.05), *p* = 0.42	0.68 (0.40–1.15), *p* = 0.15
Non-study ICUs	14.1	14.1	0		
**VAE subtypes**					
***VAC***					
Study ICUs	8.4	4.7	**↓**3.7	0.37 (0.13–1.04), *p* = 0.06	0.71 (0.28–1.78), *p* = 0.46
Non-Study ICUs	7.7	7.7	0		
***IVAC***					
Study ICUs	4.5	5.3	**↑**0.8	0.97 (0.35 – 2.70), *p* = 0.96	2.08 (0.77 – 5.58), *p* = 0.15
Non-study ICUs	4.4	4.0	**↓**0.4		
***PVAP***					
Study ICUs	1.4	0	**↓**1.4	9.42 (1.1–1238.5), *p* = 0.04	N/A
Non-study ICUs	2.1	2.4	**↑**0.3		

IRR, incidence rate ratio; OR, odds ratio. Rates are expressed as monthly events per 1,000 mechanically ventilated patient-days. Estimates in the rightmost column represent *Difference-in-Differences (DiD)* interaction effects comparing changes from the non-intervention to intervention period between study and non-study ICUs. Estimates calculated using Poisson hurdle models (for VAE/VAC/IVAC) and Firth-corrected logistic regression (for PVAP). The PVAP count model could not be estimated due to complete separation (ie, zero events in the Study ICU intervention group), so only the zero hurdle (Firth logistic) results are reported.

Among VAE subtypes, ventilator-associated condition (VAC) rates decreased in study ICUs but were unchanged in non-study ICUs, yielding a non-significant reduction within study ICUs in both the probability of any VAC (OR = 0.37, 95% CI: 0.13–1.04; *p* = 0.06) and the count among affected observations (IRR = 0.71, 95% CI: 0.28–1.78; *p* = 0.46). Infection-related ventilator-associated complication (IVAC) rates showed no consistent change across study groups, with non-significant DiD estimates in both the zero model (OR = 0.97, 95% CI: 0.35–2.70; *p* = 0.96) and the count model (IRR = 2.08, 95% CI: 0.77–5.58; *p* = 0.15). For PVAP, no events occurred in study ICUs during the intervention period, precluding estimation of a count model. In the zero model using Firth’s penalized logistic regression, the intervention period was associated with significantly higher odds of observing zero-PVAP months (OR = 9.42, 95% CI: 1.1–1238.5; *p* = 0.04), consistent with a reduction in PVAP occurrence.

## Discussion

In this *post hoc* analysis of the DIVA study, we found that implementation of a ventilator-associated pneumonia-targeted DSI was not associated with increases in VAEs or VAE subtypes—reaffirming the safety of this approach. Although diagnostic stewardship carries a theoretical risk of underdiagnosis and undertreatment, our findings show that even with substantial reductions in respiratory culture collection rates, VAE rates in study ICUs remained stable or declined. These results strengthen evidence that pneumonia-directed DSIs can be deployed safely in MVPs.^7–10^

Although the original DIVA study reported a significant post-intervention decline in VAE rates, those results were based on pre–post comparisons potentially confounded by secular trends, including the COVID-19 pandemic. In contrast, this study, which incorporated a contemporaneous comparator group and used hurdle models to account for zero inflation, did not replicate the overall VAE effect suggested by the original analyses but did suggest that the intervention was associated with reductions in PVAPs in study ICUs following DSI implementation. This decline may reflect reduced respiratory culturing—a direct target of the intervention—which in turn decreased the number of patients classified with PVAP by limiting microbiologic overdiagnosis of pneumonia.

Taken together, our findings support the safety of VAP-focused diagnostic stewardship and highlight the need for infection surveillance programs to recognize that such interventions may alter VAE subtype composition—particularly by reducing PVAP rates through decreased culture acquisition, without adversely affecting patient safety. Controlled multicenter studies are warranted to evaluate the effectiveness and generalizability of this intervention.

## Supporting information

Albin et al. supplementary material Albin et al. supplementary material

## Supplementary material

The supplementary material for this article can be found at https://doi.org/10.1017/ice.2025.10376.
