# Supplementary material for: Reductions in ventilator-associated events following implementation of a ventilator-associated pneumonia diagnostic stewardship intervention: A difference-in-difference study
Source: Infect Control Hosp Epidemiol. 2025 Dec 17;47(3):318–21. doi: 10.1017/ice.2025.10376 (PMC12932924; doi:10.1017/ice.2025.10376)
Supplement: Albin et al. supplementary material [file S0899823X25103760sup001.docx]

**Supplemental Materials**

S1. VAE Definitions

S2. Difference-in-Difference Assumptions

S3. Model Selection

S4. Coding References

**S1. Ventilator-associated event definitions**

| **VAE Tier** | **Name** | **Definition Summary** | **Required Criteria** |
| --- | --- | --- | --- |
| Tier 1 | Ventilator-Associated Condition (VAC) | Evidence of worsening oxygenation after a baseline period of stability or improvement. | • Mechanically ventilated for ≥ 4 calendar days (day of intubation = Day 1) • Baseline: ≥ 2 days of stable or decreasing FiO₂ or PEEP • Followed by ≥ 2 days of increased FiO₂ (≥ 0.20) or PEEP (≥ 3 cm H₂O) |
| Tier 2 | Infection-related Ventilator-Associated Complication (IVAC) | VAC with evidence of infection and new antimicrobial therapy. | • Meets VAC criteria AND • Temperature > 38°C or < 36°C OR WBC ≥ 12,000 or ≤ 4,000 • New antimicrobial agent initiated and continued for ≥ 4 calendar days • Criteria must occur within the VAE window period |
| Tier 3 | Possible Ventilator-Associated Pneumonia (PVAP) | IVAC with microbiologic evidence of pneumonia. | • Meets IVAC criteria AND one or more: • Positive quantitative or semi-quantitative culture from respiratory specimen • Purulent respiratory secretions AND positive culture from respiratory specimen • Positive pleural fluid or lung tissue culture, histopathology, or diagnostic testing • Note: Certain organisms (e.g., Candida spp., coagulase-negative staphylococci, Enterococcus spp.) are excluded unless recovered from lung tissue or pleural fluid |

*Legend: FiO2 = fraction of inspired oxygen, PEEP = positive end-expiratory pressure, WBC = white blood cell count.*

**S2. Difference-in-Difference assumptions**

The below table lists assumptions required for causal inference and the manner in which these assumptions were fulfilled by the study.

| *Assumption* | *Study* |
| --- | --- |
| Consistency | The study intervention was clearly defined and implemented with relatively uniform uptake and adherence across study ICUs. |
| Positivity | All ICUs at Michigan Medicine were eligible for selection into the pilot/feasibility trial. |
| Interference | There was no cross-over of trainees or staff between study and non-study ICUs, minimizing the risk of contamination. |
| Parallel Trends | A pre-intervention linear model including an interaction term between time and ICU type showed no significant interaction (p = 0.25), supporting the parallel trends assumption (see figure below). |

**
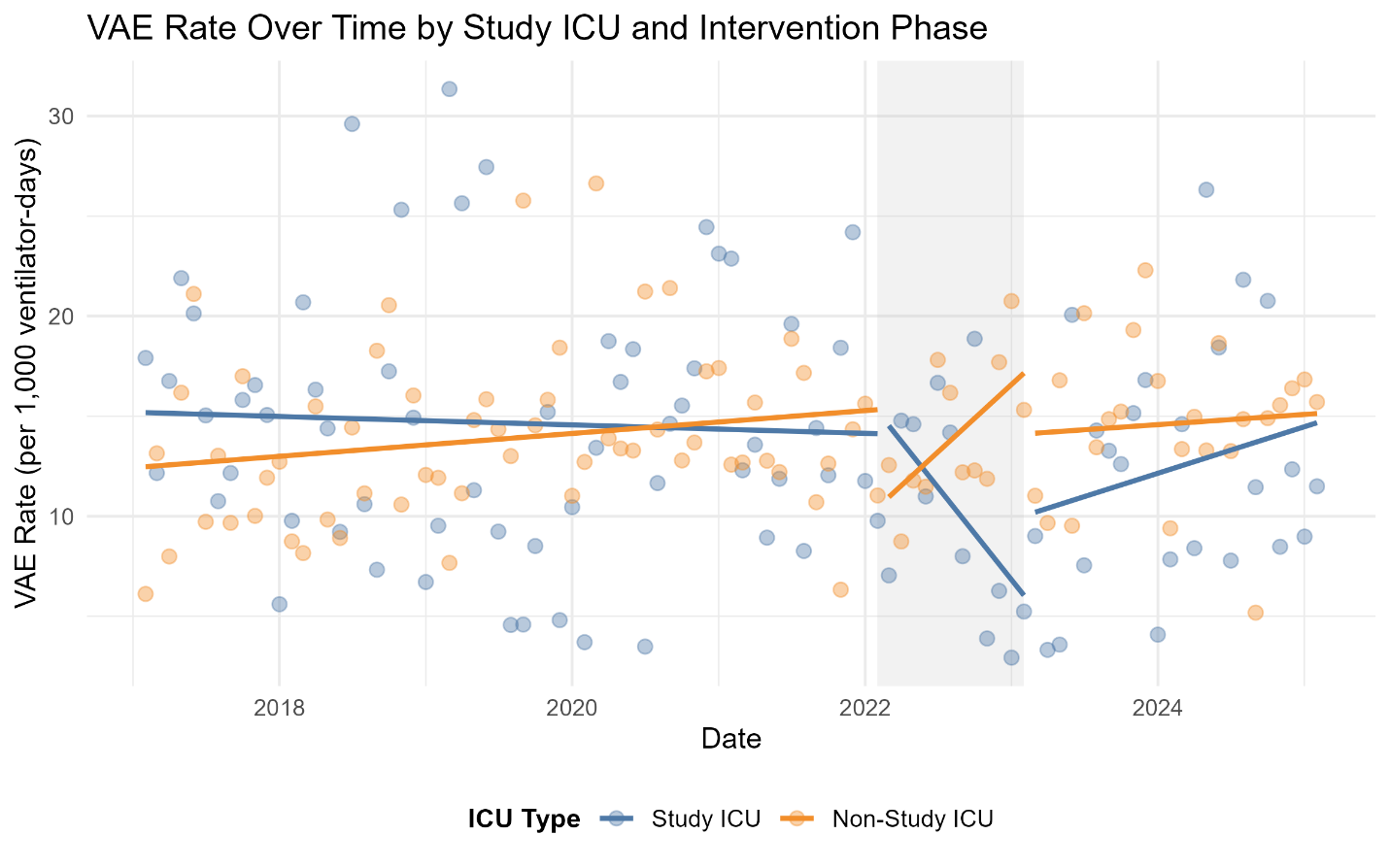
**

**S3. Model Selection**

Intraclass Correlation Coefficient (ICC) for VAE Rates (based on Poisson mixed-effects model with ICU-level clustering)

| Model | Adjusted ICC | Unadjusted ICC |
| --- | --- | --- |
| Poisson GLMM with offset | 0.033 | 0.033 |

Ljung–Box Test for Lag-1 Autocorrelation by ICU

| ICU | p-value | Significant (p < 0.05) |
| --- | --- | --- |
| 4DNI | 0.229 | No |
| 5D | 0.088 | No |
| **6D** | **0.039** | **Yes** |
| 7DN | 0.279 | No |
| **8DNS** | **0.008** | **Yes** |
| CVICU-4 | 0.109 | No |
| TBICU | 0.849 | No |

Histogram of VAE Subtypes

**
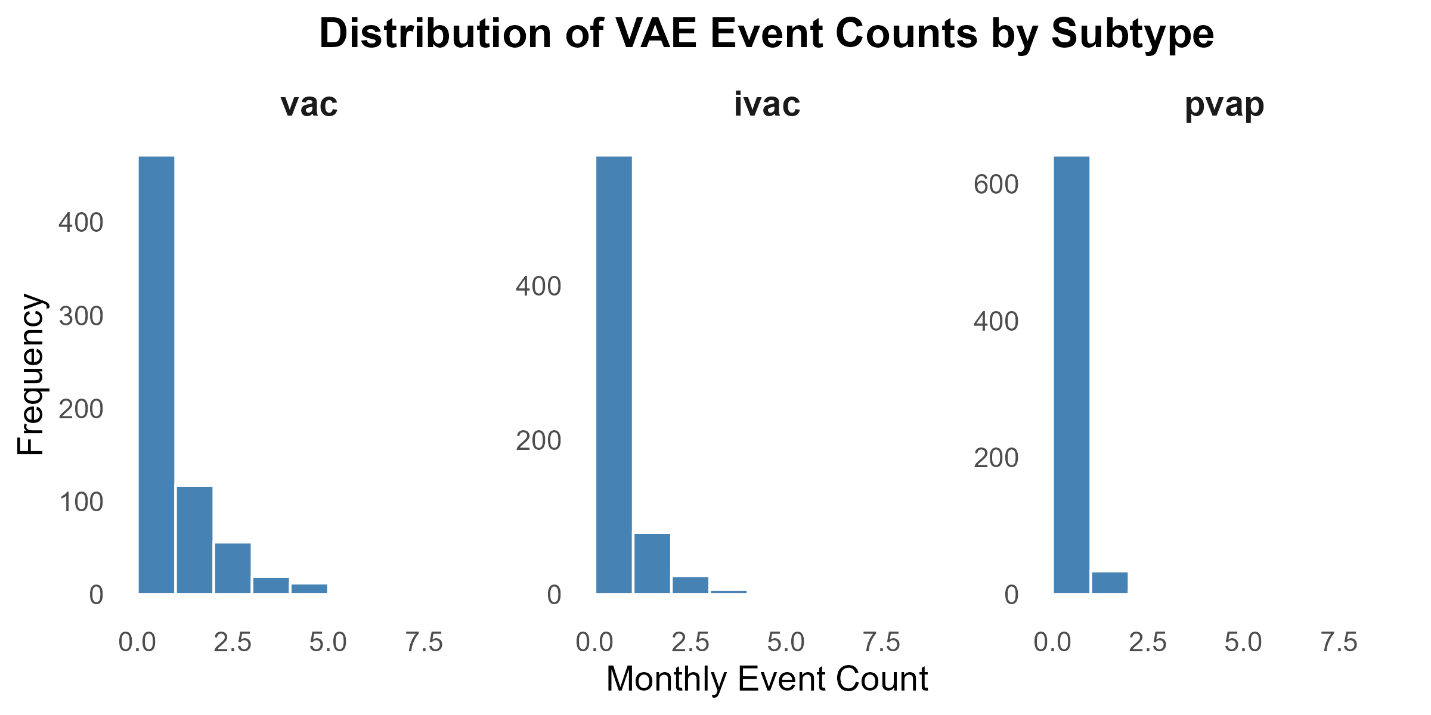
**

**S4. Coding References**

The following packages were used for data analysis within the R programming environment:

**MASS**
Venables, W. N., & Ripley, B. D. (2002). *Modern Applied Statistics with S* (4th ed.). Springer, New York. ISBN 0-387-95457-0.

**pscl**
Jackman, S. (2020). *pscl: Classes and Methods for R Developed in the Political Science Computational Laboratory.* R package version 1.5.5. https://CRAN.R-project.org/package=pscl

**logistf**
Heinze, G., Ploner, M., Jiricka, L., & Dunkler, D. (2024). *logistf: Firth’s Bias-Reduced Logistic Regression.* R package version 1.26.0. https://CRAN.R-project.org/package=logistf
Heinze, G., & Schemper, M. (2002). A solution to the problem of separation in logistic regression. *Statistics in Medicine, 21*(16), 2409–2419. https://doi.org/10.1002/sim.1047

**tidyverse**
Wickham, H., Averick, M., Bryan, J., Chang, W., McGowan, L., François, R., Grolemund, G., Hayes, A., Henry, L., Hester, J., Kuhn, M., Pedersen, T., Miller, E., Bache, S., Müller, K., Ooms, J., Robinson, D., Seidel, D., Spinu, V., Takahashi, K., Vaughan, D., Wilke, C., Woo, K., & Yutani, H. (2019). Welcome to the tidyverse. *Journal of Open Source Software, 4*(43), 1686. https://doi.org/10.21105/joss.01686

**zoo**
Zeileis, A., & Grothendieck, G. (2005). zoo: S3 Infrastructure for Regular and Irregular Time Series. *Journal of Statistical Software, 14*(6), 1–27. https://doi.org/10.18637/jss.v014.i06

**patchwork**
Pedersen, T. L. (2020). *patchwork: The Composer of Plots.* R package version 1.2.0. https://CRAN.R-project.org/package=patchwork

**glmmTMB**
Brooks, M. E., Kristensen, K., van Benthem, K. J., Magnusson, A., Berg, C. W., Nielsen, A., Skaug, H. J., Mächler, M., & Bolker, B. M. (2017). glmmTMB balances speed and flexibility among packages for zero-inflated generalized linear mixed modeling. *The R Journal, 9*(2), 378–400. https://doi.org/10.32614/RJ-2017-066

**geepack**
Halekoh, U., Højsgaard, S., & Yan, J. (2006). The R Package geepack for Generalized Estimating Equations. *Journal of Statistical Software, 15*(2), 1–11. https://doi.org/10.18637/jss.v015.i02

**clubSandwich**
Pustejovsky, J. E. (2023). *clubSandwich: Cluster-Robust (Sandwich) Variance Estimators with Small-Sample Corrections.* R package version 0.5.10. https://CRAN.R-project.org/package=clubSandwich
